# HRDepthNet: Depth Image-Based Marker-Less Tracking of Body Joints

**DOI:** 10.3390/s21041356

**Published:** 2021-02-14

**Authors:** Linda Christin Büker, Finnja Zuber, Andreas Hein, Sebastian Fudickar

**Affiliations:** Assistance Systems and Medical Device Technology, Department of Health Services Research, Carl von Ossietzky University Oldenburg, 26129 Oldenburg, Germany; linda.christin.bueker@uni-oldenburg.de (L.C.B.); finnja.zuber@gmx.de (F.Z.); andreas.hein@uni-oldenburg.de (A.H.)

**Keywords:** depth camera, timed “up & go” test, TUG, 5 × SST, marker-less tracking, machine learning, algorithm

## Abstract

With approaches for the detection of joint positions in color images such as HRNet and OpenPose being available, consideration of corresponding approaches for depth images is limited even though depth images have several advantages over color images like robustness to light variation or color- and texture invariance. Correspondingly, we introduce High- Resolution Depth Net (HRDepthNet)—a machine learning driven approach to detect human joints (body, head, and upper and lower extremities) in purely depth images. HRDepthNet retrains the original HRNet for depth images. Therefore, a dataset is created holding depth (and RGB) images recorded with subjects conducting the timed up and go test—an established geriatric assessment. The images were manually annotated RGB images. The training and evaluation were conducted with this dataset. For accuracy evaluation, detection of body joints was evaluated via COCO’s evaluation metrics and indicated that the resulting depth image-based model achieved better results than the HRNet trained and applied on corresponding RGB images. An additional evaluation of the position errors showed a median deviation of 1.619 cm (*x*-axis), 2.342 cm (*y*-axis) and 2.4 cm (*z*-axis).

## 1. Introduction

Motion capture via marker-less tracking reduces the preparation afford associated with marker-based systems such as the VICON system. Such systems can determine joint positions with an accuracy between 0.28 and 0.35 mm, depending on the density of cameras, quality of calibration, and recording conditions [1]. Among other domains, accurate motion capture systems are of special interest for gait recognition in the medical domain, where an accurate determination of joint positions enables the extraction of kinematic and kinetic gait-parameters to determine patients’ functional abilities as required to detect increased fall risks [2,3] and progression in neurocognitive diseases [4]. However, since marker-based approaches are currently too time-consuming and cost-intensive for routine use [5,6], the use of marker-less tracking-based gait analysis promises to be applied.

Thus, Fudickar et al. [7] and Hellmers et al. [8] show that the use of light barriers and/or inertial measurement units have very good results in automating the timed “up & go” (TUG) test. In addition, Dubois et al. [9] indicated the suitability to determine the number of steps and step length sufficiently accurately via depth cameras in the TUG test to distinguish fallers from non-fallers [10]. An automated sit-to-stand test can also be used to detect neurodegenerative diseases: Jung et al. [11] used a load cell embedded chair as well as a LiDAR sensor within a semi-automated implementation of the Short Physical Performance Battery to observe subjects during the 5-time-sit-to-stand test. Hellmers et al. [12] used an inertial measurement unit integrated into a belt to develop an automatic chair rise test detection and evaluation system, while Dentamaro et al. [13] classifies dementia via a developed automatic video diagnosis system for sit-to-stand phase segmentation. Yang et al. [14] also detected the gait using a single RGB camera, although this has the disadvantage that the joints can only be tracked in 2D space. Arizpe-Gomez et al. [15], on the other hand, have used three Azure Kinect cameras for automatic gait feature detection in people with and without Parkinson’s disease, but limitations here are that the data captured by Kinect is relatively noisy and at the same time the transitions between the cameras have inaccuracies.

A fundamental requirement for calculating and analyzing gait parameters with marker-less motion capture is the accurate joint-detection in still images.

Several implementations for the marker-less pose- and joint-recognition in RGB images have been proposed such as the prominent OpenPose [16] and subsequent HRNet [17]. These approaches either apply a bottom-up or a top-down approach. For the bottom-up approach, initially, keypoint positions are detected on the complete image. These keypoint positions are then fitted to complete skeletal representations per person. With the top-down approach, individual persons are initially recognized and then joint positions are detected in the corresponding image-segments. The bottom-up approach is much faster in detecting multiple persons within one image, while the detection of single persons is much more accurate via the top-down approach. These two contrary approaches are both commonly applied, as shown by the state-of-the-art joint recognition systems, OpenPose and HRNet.

In OpenPose, keypoints of body, lower and upper extremities and the head are recognized via the bottom-up approach and a convolutional neural network (CNN). Its sensitivity to detect keypoints with an average precision (AP) of 0.642 was shown for the Common Objects in Context (COCO) keypoint dataset via the evaluation metrics of COCO [16]. The evaluation metrics of COCO combine 10 metrics: average precision (AP), average recall (AR) and their variants AP^50^ and AR^50^ (AP and AR with a similarity factor of 50), AP^75^ and AR^75^ (AP and AR with a similarity factor of 75), AP^*M*^ and AR^*M*^ (AR and AR for medium-sized objects), and AP^*L*^ and AR^*L*^ (AR and AR for large-sized objects). To measure the similarity between ground-truth objects and predicted objects, an object keypoint similarity (OKS) is calculated as follows [18]:

$$\begin{array}{c} OKS=\frac{\sum_{i}\left[exp\left(\frac{-d_{i}^{2}}{2s^{2}k_{i}^{2}}\right)\delta \left(v_{i}>0\right)\right]}{\sum_{i}\left[\delta \left(v_{i}>0\right)\right]} \end{array}$$

*d_i_* is the Euclidean distance between the ground-truth and the detected keypoint position. *v_i_* indicates whether the keypoint is visible (0 for no, 1 and 2 for yes), s is the root of the area of the person for whom the keypoint was recognized, and *k_i_* = 2*σ_i_* is the per-keypoint constant that controls falloff, where *σ_i_* is 0.026, 0.025, 0.035, 0.079, 0.072, 0.062, 0.107, 0.087, and 0.089 for the nose, eyes, ears, shoulders, elbows, wrists, hips, knees, and feet. Consequently, an OKS of 1.0 indicates perfect recognized keypoints where an OKS of 0.0 indicates complete keypoint delocation [18]. The corresponding optimal results reported for HRNet and OpenPose are summarized in Table 1.

In contrast to OpenPose, HRNet applies the top-down approach via a CNN on RGB images. Due to the top-down approach, HRNet requires image sections (of 288 × 348 px) that cover only a single person instead of entire images. When evaluated under the same conditions as OpenPose, HRNet shows results significantly better than OpenPose with an AP of 0.77.

With OpenPose and HRNet focusing on the detection of poses and keypoints in the 2D RGB image plane, these approaches have been proposed for 3D human pose estimation—that is to estimate and include depth-information of keypoints. Approaches that consider depth-information typically combined this with RGB images as proposed by Véges et al. [19]. Herein, pixel coordinates of keypoints are detected via a 2D PoseNet (HRNet with a Mask-RCNN as a bounding-box detector) and the corresponding depth per keypoint coordinate is then extracted from the DepthNet (which estimates the depth per pixel). Subsequently, the data are combined to determine the 3D position per keypoint and to calculate the 3D pose from the keypoints. Evaluated via the a multi-person data set with indoor and outdoor videos (MuPoTS-3D) data set, and the Panoptic data set, consisting of several RGB-D videos captured with a Kinect, the model achieved an absolute mean position error (A-MPJPE) of 255 mm and a relative mean position error (MPJPE) of 108 mm per keypoint with a detection rate of 93%. Thereby, keypoints detection relies on evaluating RGB data, while depth information is only considered for the estimation of the depth of the joints—representing the state-of-the-art.

However, limited research has been conducted to estimate 3D keypoints solely from depth images without consideration of RGB information. Corresponding research was conducted by Ye et al. [20] who proposed the combination of body surface models and skeleton models. Herein, the depth image is initially filtered, irrelevant objects are removed, and by consideration of pre-recorded motion patterns, the poses are refined. Resulting in a mean detection error-distance for keypoints positions of 38 mm on the data set of Ganapathi et al. [21]. A similar approach was applied by Shotton et al. [22] to estimate 3D locations of skeletal joints from a single Kinect depth image. They achieved a mean average precision of 0.984 on their real test set with ground-truth labels of head, shoulders, elbows, and hands. In addition Wei et al. [23] used a single Kinect depth camera to detect and track 3D-poses simultaneously, showing an error of about 5.0 cm per joint per frame compared to the VICON system. Compared to [21,22], the system has a higher accuracy per joint (approximately between 0.85 and 1 per joint), with a deviation of up to 0.1 m to the ground truth assumed to be “correct”.

Existing approaches for human pose and joint detection require the availability of color images, RGB-D videos (i.e., color images with additional depth information), or depend on an individualized person’s data such as body surface models or skeleton models. However, with their robustness to light variation [22,24], and their color and texture invariance [22,25], as well as the easier elimination of the background [25], depth images have some advantages over RGB images. Getting the real depth value [24] also makes it possible to recognize the position of joints in 3D space, which provides further information for gait analyses or similar. However, with given advances in pose estimation and depth-cameras, the applicability of HRNet or OpenPose for depth images for marker-less tracking holds potential benefits and should be further investigated.

Correspondingly, the article at hand introduces HRDepthNet to detect keypoints of persons in depth images instead of RGB data. The model is based on the HRNet CNN model, which is retrained for annotated depth images. To evaluate the sensitivity of using an HRNet-like model for the keypoint detection via depth images instead of RGB images, the algorithm’s sensitivity is evaluated using COCO’s evaluation metrics and is compared to the sensitivity of HRNet rendered on the RGB images of the same dataset. To quantify the position error, the spatial error in cm is analyzed per keypoint.

## 2. Materials and Methods

To evaluate the suitability of pure depth images for joint recognition, we propose corresponding pre-processing (Section 2.1), a convolutional neural network based on HRNet (Section 2.2), and post-processing steps (Section 2.3). The validity to detect and locate keypoints was evaluated in a study (see Section 2.4) and a corresponding dataset is described in Section 2.5).

### 2.1. Data Preprocessing

To enhance the sensitivity of the HRNet-based model, initial pre-processing is necessary to convert the available depth information into a format suitable for model-training and use. The model expects only the image segment covering the person in a certain pixel size, but not the entire depth image. Therefore, background subtraction (via a python binding to the Point Cloud Library [26]), gray value normalization, image cropping, and scaling are conducted.

In segmentation, the area a person occupies is detected. The point cloud of the depth images is used to represent a sounder base for background elimination than depth images. As part of the segmentation procedure, smoothing, and background elimination have been applied:

For smoothing, Moving Least Squares [27] with a search radius of 0.03 was applied. For background elimination, cropping of the surroundings has been favored over general background subtraction, as we found higher robustness for variations in specific background subtraction—benefiting from the stable sensor setup and a clear path of movements. For example, background-removal of the floor was implemented via the planar segmentation algorithm with a threshold-distance of 0.02 m (as included in the PCL) which applies the random sample consensus (RANSAC) algorithm as being robust regarding outliers ([28], p. 117).

Then, the depth-axis is cropped based on the center of mass (mean z-value over all remaining points) in the *z*-axis (as representing the person) and points that exceed a threshold-distance of 50 cm from the remaining center of mass are subtracted as background.

**Figure 1:**
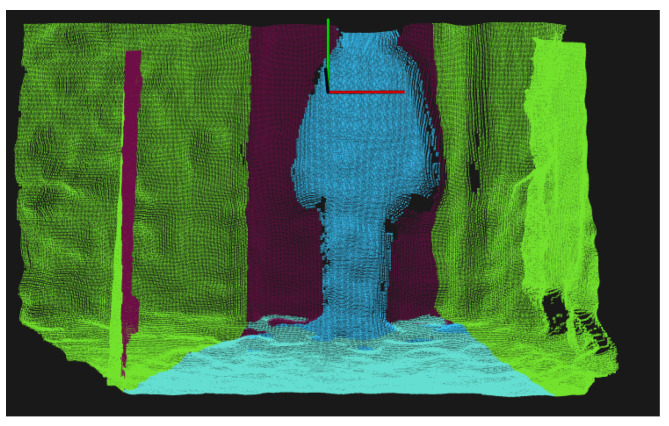
Color-coded background elimination of a point cloud. The elimination in the several axes are coded as follows: *x*-axis green, *y*-axis (floor) light blue, *z*-axis purple.

For clarity, in Figure 1 the elimination of points in the x-direction is marked in green, the elimination of the floor in light blue, and the elimination in the z-direction in purple, leaving the person marked in blue after the background elimination.

With HRNet requiring RGB images of specific proportions as input, these depth images are finally converted. From the filtered point cloud, a depth image is generated as follows: For the conversion from a point in 3D space (x,y,z) to a point on an image plane (u,v), one needs the rotation matrix R, the translation vector t, and the intrinsic matrix K [29].

$$\left[\begin{array}{c} u\\ v\\ 1 \end{array}\right]=\frac{1}{z}K\left[\begin{array}{c} R|t \end{array}\right]\left[\begin{array}{c} x\\ y\\ z\\ 1 \end{array}\right]$$

Since in this case there is no rotation and translation, the matrix $\left[\begin{array}{c} R|t \end{array}\right]$ looks like

$$\left[\begin{array}{c} R|t \end{array}\right]=\left[\begin{array}{cccc} 1 & 0 & 0 & 0\\ 0 & 1 & 0 & 0\\ 0 & 0 & 1 & 0 \end{array}\right]$$

while K, consisting of the focal length in the x and y direction ($f_{x}$ and $f_{y}$) and center of projection in the x and y direction ($pp_{x}$ and $pp_{y}$), in this case is constructed as follows:

$$\begin{array}{c} K=\left[\begin{array}{ccc} f_{x} & 0 & pp_{x}\\ 0 & f_{y} & pp_{y}\\ 0 & 0 & 1 \end{array}\right] \end{array}$$

The depth image Im is then calculated with

Imu,v=−z

*z* is multiplied by −1 for easier interpretation of depth information, since point cloud depth values are given in the negative direction.

The resulting array encodes the depth per pixel with the background being represented as 0 and the remaining depth-measures as positive values, which are encoded as gray-values, normalized to the range of [0, 255]. This normalized depth value is then encoded in a gray-scale image (see Section 2.4).

The resulting gray-scale image is further cropped and scaled under the HRNet-input requirements. From the generated gray-scale image, a section with the appropriate aspect ratio of 3:4 that covers the person and all pixels that contain depth-information >0 is extracted. This image section is then scaled to a size of 288 × 348 px representing the required image size for the input of HRNet model. The scaling factor and location of the subtraction are stored for location reconstruction in post-processing.

### 2.2. Machine Learning

For keypoint detection in depth images, transfer-learning via HRNet is the basic model for training with depth images. HRNet was preferred as the basic model for training with depth images instead of OpenPose due to these reasons: HRNet is more accurate in joint detection than OpenPose with a 15.2 higher AP. Furthermore, HRNet uses the top-down approach, which is well suited for deriving highly precise joint positions (considering corresponding raw data instead of interpolating them). This increased spatial position accuracy and the fact that in medical applications typically only a single person is visible per image, the top-down approach is more suitable for the medical-domain. For retraining, the PyTorch library version 1.4.0 of HRNet is applied with the generated depth images, heatmaps, and visible-lists (see Section 2.5). The HRNet model pose_resnet_152 with an image size of 384 × 288 is used for retraining, as the models with ResNet as backbone are more suitable for integrating them into our own system and as this explicit model showed the best results of all HRNet-models with ResNet as backbone on the COCO val2017 dataset [17]. The images are encoded as a tensor, are scaled to the interval [0,1], and normalized. For normalization, PyTorch was used with mean values (0.485, 0.456, 0.406) and standards deviations (0.229, 0.224, 0.225), as representing default-values for the ImageNet8 dataset, which is the dataset used for ResNet—the foundation of HRNet.

With the resulting normalized and scaled tensors, 20 epochs were trained and the most accurate model over 20 epochs was finally selected. Per epoch, the batch composition was randomized. The adaptive moment estimation (Adam) optimizer was used [30]. For loss calculation JointsMSELoss was chosen, which calculates the average of the individual mean squared error (MSELoss, suitable for regression challenges as is the given one) of all visible keypoints among the reference and detected annotation:

$$\begin{array}{c} JointsMSELoss=\frac{\sum_{n}0.5\times MSELoss\left(joint_{n}\right)}{n},\mathrm{with}n=\mathrm{number}\;\mathrm{of}\;\mathrm{joints} \end{array}$$

For evaluation, ten models are trained.

### 2.3. Post-Processing: Keypoint Visibility

The resulting ML model’s heatmap encodes per coordinate the probability to hold a keypoint. Per keypoint, the location with the highest probability is chosen. As the heatmap encodes as well invisible keypoints (e.g., that are hidden by other body-parts), separate filtering is applied to remove invisible keypoints from the evaluation. Only keypoints that surpass a probability-threshold of 0.83 are accepted as valid and all others are rejected as invisible.

**Figure 2:**
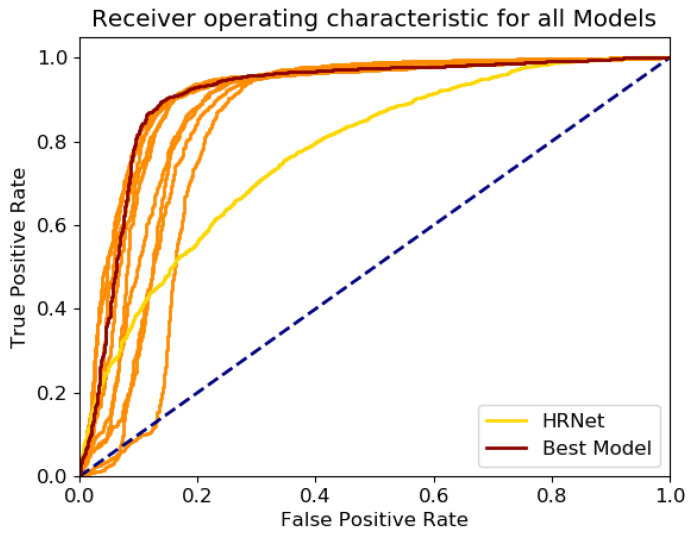
ROC-curves of the models. The best model in red and the HRNet in yellow.

The corresponding threshold was determined as the optimal combined true positive rate ($tpr_{t}$) and false positive rate ($fpr_{t}$), which was determined as the distance $dist_{t}$ per threshold value *t* (see Figure 2) to the point tpr = 1, fpr = 0.

$$\begin{array}{c} dist_{t}=\left|\left(1,0\right)\left(tpr_{t},fpr_{t}\right)\right| \end{array}$$

The used threshold *t* was determined as holding minimal distance $dist_{t}$.

In addition, the location of the keypoints on the original images is reconstructed from the cropped and scaled depth image via the saved scaling factor and location of the clipping window.

### 2.4. Study Design

For training and evaluation of the proposed HRDepthNet, a corresponding training and evaluation dataset was generated in a study. The study considers humans conducting the TUG test. The TUG test is an established geriatric functional assessment which includes activities such as walking, turning, and sitting and corresponding transitions in between these. This assessment covers multiple critical body-postures prone to increased classification errors [31].

**Figure 3:**
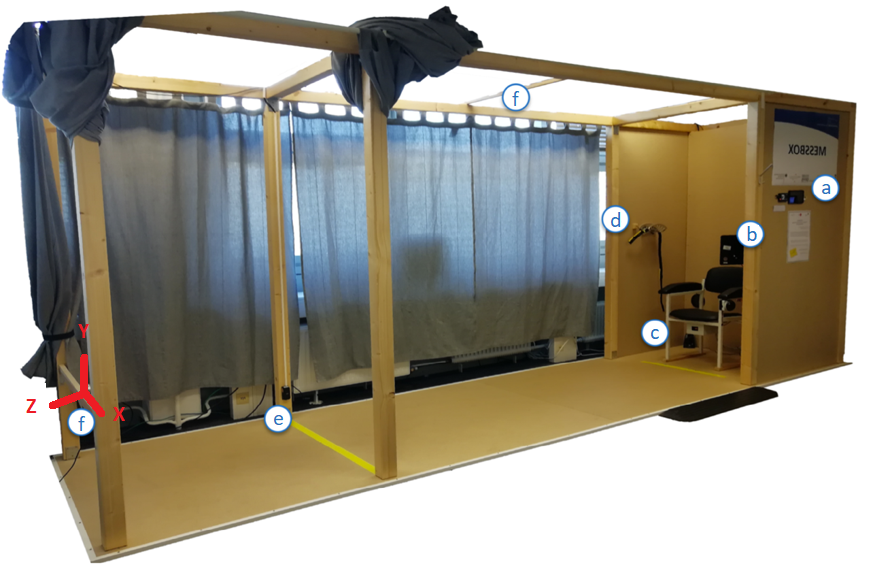
The Unsupervised Screening System (USS) consisting of: (**a**) an introductory display with a RFID authentication device, (**b**) a main display for user-interaction, (**c**) an integrated automated TUG chair with light barriers, (**d**) a sensor-belt with an included inertial sensor, (**e**) light barriers and a yellow line at a 3 m distance and (**f**) Intel RealSense^TM^ D435 depth image cameras (one placed approximately 4 m in front of the chair and one facing the chair from the upper level). Relevant items for this study are (**c**,**e**,**f**).

The recording took place in the Unsupervised Screening System (USS) [7], shown in Figure 3.

The USS includes a chair (c), where subjects start and finish each test-run in a seated position. Crossing a yellow line (e), 3 m in front of the chair, the subjects were expected to turn around and head back to the chair following the TUG test-protocol.

**Figure 4:**
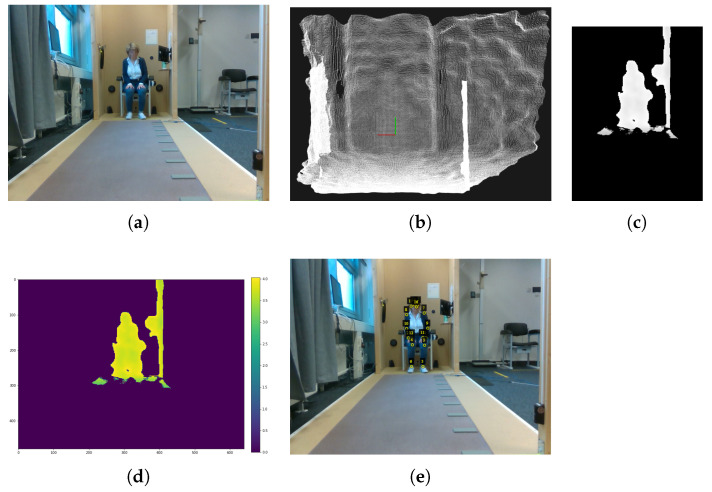
Example images of the RAW (**a**) RGB image (**b**) point cloud, (**c**) depth image, (**d**) color coded representation of the depth image, and (**e**) keypoint annotations.

The test-execution was recorded using an Intel RealSense^TM^ D435 RGB-D camera (f), which was placed at knee height 4.14 m in front of the chair facing towards the participants and the chair (see Figure 4b). Depth images are recorded via an active infrared stereo sensor. Both, RGB and depth images were captured with 640 × 480 px resolution at 30 fps and recordings were stored in .bag files. The sensor orientation (see Figure 3) sets the *x*-axis horizontal, the *y*-axis vertical, so that the *z*-axis is covering the depth (as the distance from the sensor). Depending on calibration-settings and ambient light conditions, the reported distance of the depth-sensor ranges from 20 to 1000 cm. The RGB-D camera was connected to a PC, running a Python-script for starting and stopping recordings and data-storage.

Participants that met the following inclusion criteria (adults that have no neurological or psychiatric disorders, can walk 6 min without a break, and have full or corrected vision) were considered and invited to participate. Participants were informed about the study procedure and signed informed consent (in accordance with Declaration of Helsinki [32]). Their sex, age, height (cm), weight (kg), leg length (cm), and pre-existing conditions related to mobility were collected.

Participants conducted the TUG test repetitively at varying walking paces (normal, fast, and slow) each at least five repetitions per pace and were asked to include breaks as needed. Pace variations were subject to personal perception. The repetition of these test-blocks was extended depending on participants’ willingness and capacity and was ended latest after maximal 2 h. Per TUG execution, a new recording was initialized.

As no multi-person view is considered yet, images intentionally cover only single persons. By covering most common activities and including a varying distance of 1 to 4.2 m from the camera, facing the persons’ front, the corresponding recordings make a well-suited training and evaluation dataset.

The study was approved by the Commission for Research Impact Assessment and Ethics at “Carl von Ossietzky University of Oldenburg” (Drs.EK/2019/094) and was conducted in accordance with the Declaration of Helsinki [32].

### 2.5. Preparation of the Dataset

As no suitable dataset is yet available, we created a dataset as follows. While the captured RGB-D videos were recorded with 30 Hz, subsequent video-frames are remarkably similar regarding keypoint-position. However, HRDepthNet is not intended to track keypoints but to detect the keypoint-positions in still images. For this reason, a high frame rate is not necessary and consecutive video-frames have only limited benefit for model training and evaluation. Thus, a frequency of 5 Hz was found sufficient and only every sixth video-frame is considered in the dataset. In addition, the initial frames per shot were discarded since the camera’s exposure control takes milliseconds to adjust to lighting situations and correct manual recognition of joint positions was impossible for them. From the considered frames of the RGB-D videos, depth images were extracted as input for keypoint detection, while corresponding RGB images were used for annotation and HRNet evaluation.

From the depth-videos, individual images were extracted, aligned with the RGB images (via align and process commands), and stored as point clouds via the pyrealsense2 library. In addition to the pre-processing of the depth images (see Section 2.1), RGB image size is scaled to the depth images and RGB images are mirrored along the *y*-axis. By correcting lens-distortions among both image types, the validity of keypoints coordinates for the depth images is ensured.

Images are annotated regarding the actual keypoint positions and the person overlapping polygons (as required input for the COCO evaluation metric). Keypoint positions were manually annotated in the two-dimensional coordinate system via the VGG Image Annotator (VIA) software [33] and visual inspection based on RGB images and guidelines for keypoint positioning as described in Section 1. Thereby, using RGB images instead of depth images for annotation ensures greater positioning accuracy. This is because keypoint recognition is more challenging with depth images for human annotators—especially at greater distances from the camera—due to a reduced signal-to-noise ratio. To increase the efficiency of the annotation process, keypoint positions in RGB images were pre-labeled using the HRNet model, transferred into a VIA conform file-format, and then were adjusted manually via VIA. Afterwards, the corrected 2D keypoint positions were converted into HRNet conform heatmaps. These heatmaps are generated per visible keypoint, under HRNet. Per keypoint, a 2D Gaussian Function with a standard deviation of 1 pixel is run from the annotated keypoint coordinate. A visible-list is generated per image. In the visible-list, all keypoints, which are annotated are encoded binary as 1.

The COCO metric requires for evaluating the keypoint similarity also a specification of the area occupied by humans per image (which is typically estimated via the polygon of their contour). Thus, this contour was also annotated manually as a polygon in the RGB images via the VIA software and visual inspection, and polygons and the outlined areas are stored COCO conform in a JSON file.

Images were grouped in approximately 70% training-set, 15% validation-set, and 15% test-set. For the test-set, images of a randomly selected subject were selected exclusively (leave one out).

### 2.6. Analysis

To achieve comparability among results, evaluating the proposed approach considers the evaluation metrics of COCO [34]. Since evaluating the keypoint accuracy by mimicking the evaluation metrics used for object detection, these metrics include average precision (AP), average recall (AR) (both including AP^*M*^/AR^*M*^ and AP^*L*^/AR^*L*^ and for medium and large person representations), and the corresponding second and third quantiles and object keypoint similarity (OKS).

To investigate the generalizability of the approach, ten models have been trained and evaluated via these metrics. For comparability to the original HRNet (pose_hrnet_w48 with image size 384 × 288), which showed the best results of all HRNet-models on the COCO val2017 dataset [17], it was evaluated on the corresponding dataset’s RGB images. As the COCO evaluation metric evaluation of OKS only considers the spatial correctness of the visible keypoints in the ground-truth, it does not consider the correct detection of non-visible keypoints. To evaluate the suitability of the proposed threshold-based filtering of non-visible keypoints, we first generated receiver operating characteristic (ROC) curves to identify the rate of TP and FP for each of the ten generated ML models and to determine the best threshold for each model, which has been used for these analyses. The ROC-curve has been also plotted for the HRNet on the dataset’s RGB images.

To determine the most accurate model, we evaluated the sensitivity and specificity by which the key-points are correctly detected (as visible or invisible) via the F1-score. The F1-score is calculated as follows with precision (*p*) and recall (*r*):

$$\begin{array}{c} f_{1}=2\times \frac{p\times r}{p+r} \end{array}$$

In addition to the mean F1-score for all keypoints, as required for model selection, we also calculated keypoint-specific F1 scores.

Further evaluation was conducted only on the best among the 10 models. For evaluation of the validity to detect only visible keypoints, a corresponding confusion matrix was calculated.

For the consideration of individual keypoints, true-positive and true-negative rates and associated parameters are evaluated on a per-joint level. In addition, to analyze the accuracy of the keypoint-positions, deviations were mapped to reference-labels. Only visible keypoints are considered by the model. In addition, joints with invalid depth values, as indicated by the sensor reading of zero, have been excluded to overcome these consequentially erroneous positions.

Per (TP) keypoint-location median spatial errors are calculated per axis and corresponding boxplots have been generated.

## 3. Results

### 3.1. Study Sample

Eight subjects aged between 23 and 55 years (5 female, 3 male) with body-heights ranging from 160 to 193 cm and weights ranging from 57 to 108 kg participated in the study. A total of 293 videos with an in total duration of 54 min and 23 s have been recorded. Among these, 35 videos with an overall duration of 6 min and 40 s that are equally and randomly distributed among the eight subjects were considered. Allover, 1970 images were included and annotated—leading to 224 to 264 images per participant; thus, equal distribution among participants is achieved. Distributing these images among the training- and development-set was 1349 images (training) (68.48%) and 357 images (dev) (18.12%). The subject for evaluation-set covered 264 images (13.4%).

### 3.2. Keypoint Detection Accuracy

Table 2 summarizes the results of the 10 trained models in comparison to the HRNet on the RGB images. The results indicate that the HRDepthNet models perform better or at least like the HRNet model. The average results of the trained models are always higher than those of the HRNet when considering all keypoints. Only the minimum AP^50^ is below the result of the HRNet (0.011 difference).

For lower extremities (hips, knees, ankles) the accuracy of all HRDepthNet models surpasses the HRNet in general. The average AP is 0.247 and the average AR 0.232 higher than the HRNet.

For upper extremities (nose, eyes, ears, shoulders, elbows, wrists), mean results are comparable among HRDepthNet and HRNet. With the worst result of the 10 trained models always being below the HRNet and, on the other hand, the best results of the 10 trained models, surpassing the HRNet.

### 3.3. Sensitivity among Models

The ROC curves of the 10 trained HRDepthNet models are shown in Figure 2 with the best model (regarding the fitting towards the 0.83 threshold) and thus further on selected model (1) in red and the HRNet in yellow.

Table 2 summarizes the COCO metrics and the model’s performance, as measured via the mean, minimal, and maximal errors for all models.

The calculated F1 score, precision, and recall per model (as applied subsequently to filtering keypoints via thresholding) are summarized in Table 3. As model 1 has the highest combined sensitivity and specificity (as F1 score) regarding correctly detecting the visibility of keypoints with the proposed filtering threshold, subsequent evaluations are only conducted for model 1.

The sensitivity of the best model in detecting visible and occluded keypoints/joints with the threshold filter applied is shown in the corresponding confusion-matrix in Table 4. The results indicate 88% of visible and 87% of invisible joints are correctly classified.

### 3.4. Consideration of Individual Keypoints

To evaluate the suitability of HRDepthNet to detect keypoints in specific body-regions, Table 5 summarizes the accuracy metrics of individual keypoints of the best model. While most keypoints and especially the ones of the head are well detected, the detection of the right hip and the left ear is less accurate (as indicated by lower TP and TN rates and F1-score).

**Figure 5:**
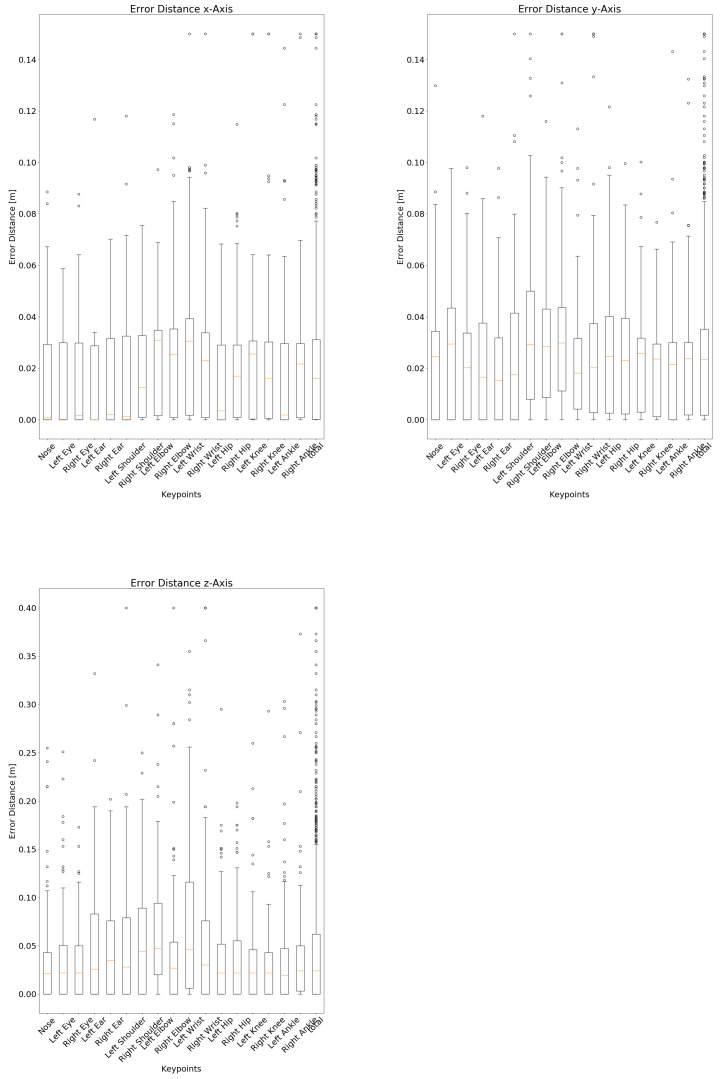
Box plots of spatial error-distances of detected keypoints (with valid depth-information) compared to annotated positions (as reference) per axis and keypoint. Plots have been chopped at 0.15 m (lateral x and height y-axis) and 0.4 m (depth z-axis) and corresponding outliers have been aggregated at the upper range.

In addition to the classification accuracy of the keypoints, an essential aspect is the spatial accuracy of the correctly detected (true positive) keypoints, as compared to the reference annotations. Thereby, joints with invalid depth values have been excluded (10 joints in total, 0.4% of all considered keypoints). The box plots in Figure 5 show keypoint-specific spatial errors from reference. Corresponding keypoint-specific median spatial errors are as well summarized in Table 5. Each joint has been accurately detected with a deviation of 0 cm at least once. The largest deviation of 0.903 m on the *x*-axis, 1.211 m on the *y*-axis, and 3.405 m on the *z*-axis (axis alignment in Figure 3) all occur for the same joint in the same image, so they can be exceptions, as found via visual inspection.

## 4. Discussion

### 4.1. Sensitivity of Depth Image-Based HRNet

To address the research question how accurate is the keypoint detection via depth images compared to RGB images, we evaluated the accuracy to detect keypoints via the HRDepthNet for depth images and the HRNet via RGB images for the given datasets and made these findings:

The originally reported average APs for HRNet (0.77) and OpenPose (0.64) for the COCO dataset slightly surpassed the results for HRNet in our dataset (0.61) (compare Table 1 and Table 2). This might be due to the fact both have been trained on an alternative dataset where multiple subjects are covered and keypoints might be less frequently obstructed.

All average APs and ARs of the HRDepthNet surpassed those of HRNet, except for average AP^50^ for the upper extremities (see Table 2). Corresponding maximum APs and ARs for HRDepthNet were in general higher than the corresponding ones of HRNet. Thus, we found that joint detection based on depth images can be achieved with at least similar accuracy than with RGB images. Typically, an increase of the AP by 0.06–0.11 and of the AR by 0.1 can be expected.

Considering the variations among the trained HRDepthNet models, the F1-scores in Table 3 and the corresponding ROC-curves in Figure 2 confirm a low variability among the HRDepthNet models and justify the generality of our further findings which focus on only the best HRDepthNet model. The higher AUC of the ROC curves of all HRDepthNet models compared to the AUC of the HRNet indicates higher suitability of the HRDepthNet especially regarding their steeper increase and thus a significantly lower false-positive rate (best thresholds show an fpr of 0.13 on the best model and a fpr of 0.3 on the HRNet).

The high suitability of the best HRDepthNet model to differentiate between visible and occluded keypoints by applying the threshold is shown in the confusion matrix in Table 4. With 88% of visible and 87% of occluded keypoints being correctly classified as such, the risk of considering occluded keypoints, which might increase the spatial error is sufficiently addressed. Instead of considering occluded keypoints, we foresee a benefit to interpolate missing keypoints from surrounding frames and achieving a sufficient accuracy for further time series-based analysis.

### 4.2. Specific Consideration of Keypoints

For a specific consideration of specific keypoints, we investigated the effect of subject size and studied the accuracy for body-regions and specific keypoints. Considering the differentiated evaluation of medium- and large-sized subjects, both HRNet and OpenPose have found an increased AP for medium-sized subjects (0.097 and 0.08, respectively). These results were confirmed as HRNet when applied on our dataset achieved an increased AP of 0.053 for medium-sized subjects. In addition, HRDepthNet achieved an average increase of AP of 0.021 for medium-sized subjects. The increase was more prevalent for the upper and lower extremities, where HRDepthNet achieved an AP increase of 0.1 of upper- and 0.15 for the lower extremities among both groups. The error of HRNet in these body-regions is even higher.

Comparing the accuracy of HRNet and HRDepthNet for detecting keypoints of the upper and lower extremities (Table 2), we found a higher accuracy of HRDepthNet for the lower extremities (with an increase of AP by 0.22 and AR by 0.16), while HRNet was slightly more accurate for the upper extremities (with an increase of AP by 0.009 and an increase of AR of 0.055).

HRDepthNet’s body-part specific F1-scores, shown in Table 5 indicate comparable accuracy in differentiating among visible and occluded keypoints. With most of the keypoint-specific F1-scores remaining above 0.88, classification of the left ear and the right hip experienced a relevant drop of F1-scores with 0.67 and 0.82, respectively. For the hip, the error might be even caused by the challenge to correctly locate the hip-position under clothing during annotation—as representing, in general, the most challenging joints and keypoints. The left ear’s low F1-score was mainly caused by subjects having longer hair covering the left ear than the right one and the left ear being less frequently visible on the recordings and therefore as well in the training set.

As the most relevant for the extraction of gait-parameters, the lower extremity keypoints (ankle and knee) deserve special interest. The visibility of both ankle joints and knee joints were correctly detected with sufficient accuracy and an F1-score between 0.89 and 0.91. Thus, we expect suitability of HRDepthNet for gait analysis to be confirmed in a subsequent article.

### 4.3. Spatial Accuracy

In addition to the ability to correctly detect keypoints and differentiate among visible and occluded keypoints, the spatial accuracy by which the joints can be localized is essential for application in the medical domain. Thus, to clarify which spatial errors can be expected from joint detection via depth images, we as well investigated the spatial error by which each keypoint is localized per axis (recap the axis alignment in Figure 3). The corresponding boxplots are shown in Figure 5 and median error-distances in Table 5. With median errors for the lateral and height axis remaining well within 3.1 cm, errors for the some of the upper-extremities’ joints on the depth-axis were up to 4.7 cm higher. However, with median errors below 2.6 cm, joints of the lower extremities were slightly more accurate than the upper extremities. Comparing the distance among the averaged axis-specific errors of the proposed approach with the errors reported in the literature, the approach presented here performs very well. Thus, the mean error-distance of Véges et al. [19] (255 mm A-MPJPE), Ye et al. [20] (38 mm mean detection error-distance), and Wei et al. [23] (5 cm error per joint per frame) are all above the distance between the averaged axis-specific errors of the proposed approach, which measures 3.724 cm.

The boxplots as well indicate outliers for all keypoints, which can be reduced by interpolating the positions among subsequent video-frames as an additional post-processing step. Among all keypoints, outliers and limits are equally distributed.

With convincing overall spatial accuracy, the results of the depth-axis need further consideration. Since the error on the depth-axis for the used sensor includes a measurement error of 2–4 cm [35] and no reference system was considered, its reported spatial error only represents errors resulting from misalignment of the keypoint position. A larger depth error indicates a keypoint that lies beside the subject and measures background locations. We intend to evaluate the correctness in depth more thoroughly in an upcoming study by comparing the sensitivity to a VICON reference-system.

## 5. Conclusions

The article introduces HRDepthNet for joint detection in depth images. It extends on HRNet by retraining it on depth images. HRDepthNet was trained via a new dataset including depth images and corresponding RGB of moving persons conducting the timed “up & go” test—an established functional for geriatric patients. With this dataset, we evaluated the accuracy of HRDepthNet and HRNet via the COCO metrics. The results indicate that HRDepthNet shows similarly reliable results as the original HRNet trained on RGB images. The lower joints even show better results than the HRNet, while the upper key points show worse results.

We evaluated HRDepthNet’s accuracy in discriminating between visible and occluded keypoints, showing that by applying a threshold-based filtering an F1-score of around 90% can be achieved. In addition, the spatial accuracy was evaluated indicating a maximum median error below 5 cm for all keypoints. With median errors below 2.6 cm, keypoints of lower extremities were located even more accurately. The article indicates that the use of depth images instead of RGB images for joint detection is a suitable alternative. The current article has the following limitations: Even though the study sample was well distributed in age, gender, size, and weight and are well suited for the study questions, inclusion of further subjects will enhance the reliability of the findings. In addition, the considered images intentionally cover only single persons as being representative for applications in the medical domain. Thus, findings regarding the accuracy will vary for conditions with multi-persons. To further on as well study the approaches’ medical applicability, further processing steps (such as pose tracking) are an essential progression, to be considered in upcoming articles.

## Figures and Tables

**Table 1:** COCO evaluation metrics for OpenPose and HRNet on the COCO test-dev dataset.

	AP	AP^50^	AP^75^	AP^*M*^	AP^*L*^	AR
OpenPose	0.642	0.862	0.701	0.61	0.688
HRNet	0.77	0.927	0.845	0.734	0.831	0.82

**Table 2:** Results of the COCO metrics among the 10 trained models on the depth images compared to the HRNet w48 384 × 288 when applied to the RGB images of the dataset. Separately for all 17 keypoints, keypoints of upper extremities (nose, eyes, ears, shoulders, elbows, wrists) and keypoints of lower extremities (hips, knees, ankles).

	AP	AP^50^	AP^75^	AP^*M*^	AP^*L*^	AR
All keypoints						
Average	0.725	0.919	0.846	0.756	0.735	0.783
Min	0.692	0.887	0.812	0.736	0.693	0.747
Max	0.755	0.939	0.893	0.778	0.775	0.807
HRNet	0.61	0.898	0.648	0.693	0.62	0.669
Upper Extremities						
Average	0.633	0.906	0.705	0.713	0.61	0.724
Min	0.579	0.862	0.648	0.682	0.545	0.671
Max	0.669	0.928	0.738	0.754	0.65	0.753
HRNet	0.618	0.919	0.692	0.738	0.58	0.706
Lower Extremities						
Average	0.856	0.933	0.881	0.974	0.826	0.883
Min	0.819	0.904	0.843	0.964	0.777	0.869
Max	0.884	0.95	0.909	0.983	0.862	0.898
HRNet	0.609	0.832	0.594	0.714	0.578	0.651

**Table 3:** Precision, recall, and F1-score for all models after applying the threshold. The model with the best F1-score is written in bold.

Model	Precision	Recall	F1-Score
**1**	**0.92**	**0.882**	**0.901**
2	0.916	0.88	0.897
3	0.884	0.88	0.882
4	0.86	0.883	0.872
5	0.872	0.88	0.876
6	0.909	0.88	0.894
7	0.919	0.88	0.899
8	0.909	0.881	0.895
9	0.883	0.88	0.881
10	0.914	0.88	0.897

**Table 4:** Confusion matrix for differentiating visible and occluded keypoints via the best HRDepthNet model.

		Classified Keypoints	
		Visible	Occluded
Reference Keypoints	visible	2488 (88%)	332
	occluded	217	1451 (87%)

**Table 5:** The number of keypoints correctly and incorrectly recognized as visible by the best model with a threshold value and the right-positive and right-negative rate per joint. Sensitivity = TP/(TP + FN); specificity = TN/(TN + FP); the error-distances have been calculated for the filtered keypoints.

								Median Error Distance [cm]		
Keypoints	TP	FP	FN	TN	Sensitivity	Specificity	F1-Score	Lateral (x)	Height (y)	Depth (z)
Nose	113	12	2	137	0.983	0.919	0.942	0.078	2.446	2.1
Left Eye	99	17	0	148	1	0.897	0.921	0.005	2.948	2.2
Right Eye	104	17	1	142	0.99	0.893	0.92	0.162	2.019	2.2
Left Ear	51	49	1	163	0.981	0.769	0.671	0.002	1.658	2.6
Right Ear	118	14	1	131	0.992	0.903	0.94	0.209	1.527	3.5
Left Shoulder	123	13	4	124	0.969	0.905	0.935	0.122	1.754	2.8
Right Shoulder	141	6	6	111	0.959	0.949	0.959	1.261	2.935	4.4
Left Elbow	154	12	17	81	0.901	0.871	0.914	3.09	2.846	4.7
Right Elbow	166	7	23	68	0.878	0.907	0.917	2.543	2.987	2.7
Left Wrist	155	17	17	75	0.901	0.815	0.901	3.054	1.815	4.6
Right Wrist	182	8	42	32	0.813	0.8	0.879	2.305	2.026	3.0
Left Hip	168	12	23	61	0.88	0.836	0.906	0.343	2.454	2.2
Right Hip	159	19	48	38	0.768	0.667	0.826	1.693	2.296	2.2
Left Knee	191	5	34	34	0.849	0.872	0.907	2.558	2.579	2.2
Right Knee	197	2	39	26	0.835	0.929	0.906	1.619	2.357	2.2
Left Ankle	178	6	39	41	0.82	0.872	0.888	0.182	2.138	1.95
Right Ankle	189	1	35	39	0.844	0.975	0.913	2.167	2.365	2.4
Upper Extremities	1406	172	114	1212	0.925	0.876	0.908	1.68	2.296	2.7
Lower Extremities	1082	45	218	239	0.832	0.841	0.892	1.537	2.371	2.2
Total	2488	217	332	1451	0.882	0.87	0.901	1.619	2.342	2.4

## Data Availability

Data sharing is not applicable to this article.
